# Prophylactic Supplementation of *Bifidobacterium longum* 5^1A^ Protects Mice from Ovariectomy-Induced Exacerbated Allergic Airway Inflammation and Airway Hyperresponsiveness

**DOI:** 10.3389/fmicb.2017.01732

**Published:** 2017-09-11

**Authors:** Eduardo Mendes, Beatriz G. Acetturi, Andrew M. Thomas, Flaviano dos S. Martins, Amanda R. Crisma, Gilson Murata, Tárcio T. Braga, Niels O. S. Camâra, Adriana L. dos S. Franco, João C. Setubal, Willian R. Ribeiro, Claudete J. Valduga, Rui Curi, Emmanuel Dias-Neto, Wothan Tavares-de-Lima, Caroline M. Ferreira

**Affiliations:** ^1^Department of Pharmaceutics Sciences, Institute of Environmental, Chemistry and Pharmaceutical Sciences, Universidade Federal de São Paulo Diadema, Brazil; ^2^Department of Pharmacology, Institute of Biomedical Sciences I, University of São Paulo São Paulo, Brazil; ^3^Medical Genomics Laboratory, CIPE/A.C.Camargo Cancer Center São Paulo, Brazil; ^4^Department of Biochemistry, Institute of Chemistry, University of São Paulo São Paulo, Brazil; ^5^Bioinformatics Graduate Program, University of São Paulo São Paulo, Brazil; ^6^Department of Microbiology, Institute of Biological Sciences, Federal University of Minas Gerais Belo Horizonte, Brazil; ^7^Department of Physiology and Biophysics, Institute of Biomedical Sciences, University of São Paulo São Paulo, Brazil; ^8^Department of Immunology, Institute of Biomedical Sciences IV, University of São Paulo São Paulo, Brazil; ^9^Post Graduate Program in Biophotonics Applied to Health Sciences, Universidade Nove de Julho São Paulo, Brazil; ^10^Department of Pharmacy and Biotechnology, Universidade de Anhanguera de São Paulo São Paulo, Brazil; ^11^Laboratory of Neurosciences (LIM-27), Institute of Psychiatry, Medical School University of São Paulo São Paulo, Brazil

**Keywords:** probiotic, *Bifidobacterium longum*, airway inflammation, ovariectomy, microbiota

## Abstract

Asthma is a chronic inflammatory disease that affects more females than males after puberty, and its symptoms and severity in women change during menstruation and menopause. Recently, evidence has demonstrated that interactions among the microbiota, female sex hormones, and immunity are associated with the development of autoimmune diseases. However, no studies have investigated if therapeutic gut microbiota modulation strategies could affect asthma exacerbation during menstruation and menopause. Here we aimed to examine the preventive effects of a probiotic, *Bifidobacterium longum* 5^1A^, on airway inflammation exacerbation in allergic ovariectomized mice. We first evaluated the gut microbiota composition and diversity in mice 10 days after ovariectomy. Next, we examined whether re-exposure of ovariectomized allergic mice to antigen (ovalbumin) would lead to exacerbation of lung inflammation. Finally, we evaluated the preventive and treatment effect of *B. longum* 5^1A^ on lung inflammation and airway hyperresponsiveness. Our results showed that whereas ovariectomy caused no alterations in the gut microbiota composition and diversity in this animal model, 10 days after ovariectomy, preventive use administration of *B. longum* 5^1A^, rather than its use after surgery was capable of attenuate the exacerbated lung inflammation and hyperresponsiveness in ovariectomized allergic mice. This prophylactic effect of *B. longum* 5^1A^ involves acetate production, which led to increased fecal acetate levels and, consequently, increased Treg cells in ovariectomized allergic mice.

## Introduction

Asthma is a chronic inflammatory lung disease and a serious health concern (Cohn et al., 2004; Leynaert et al., 2012). The number of people with asthma worldwide increased from 235 to 334 million between 2011 and 2014 (Ellwood et al., 2017). At age 11, the prevalence of asthma is greater in males than in females; however, after puberty, this trend reverses. Furthermore, in adulthood, the mean duration of hospital stay is longer for women than for men, suggesting not only that the prevalence is greater in women after puberty but also that the disease is more severe (Hanley, 1981; Gibbs et al., 1984; Eliasson et al., 1986). Clinical evidence shows that a significant percentage of women with asthma exhibit worsening of symptoms during the perimenstrual phase (shortly before and during the first few days of the menstrual period). Terms such as (pre)menstrual, perimenstrual or circamenstrual asthma have been used to describe this phenomenon. It has been suggested that hormonal fluctuations during the menstrual cycle play a significant role in the periodic worsening of asthma severity in adult females. The hormonal fluctuations that occur in menopause can also trigger or exacerbate adult-onset asthma (Troisi et al., 1995; Bellia and Augugliaro, 2007; Zemp et al., 2012; Triebner et al., 2016). The roles of gender and sex hormones in asthma are very complex. Recently, experimental findings have demonstrated that interactions among the microbiota, female sex hormones, and immunity are associated with the development of autoimmune diseases (Markle et al., 2013; Yurkovetskiy et al., 2013). In this scenario, it has been clearly recognized that germ-free NOD female mice lack the commonly observed gender bias of diabetes, with enhanced type 1 diabetes development found in these females, showing a pivotal role of the gut microbiota in disease development. On the other hand, after castration, male mice exhibit a microbiota composition that is more similar to that found in female mice. Overall, these data demonstrate that female sex hormones, rather than X chromosome-associated factors, are involved in the modulation of microbiota composition (Markle et al., 2013; Yurkovetskiy et al., 2013). Thus, it is strongly suggested that hormones and the microbiota cooperate to modify the course of disease progression. Given the recognized role of the gut microbiota in regulating the immune response via hormone interactions, it is not surprising that the oscillating hormone levels observed during the menstrual cycle and in menopausal women could interfere with the gut microbiota profile, which in turn may affect female asthma symptoms.

The most popular probiotics are lactobacillus and bifidobacteria. Probiotic strains such as *Lactobacillus reuteri* (Miraglia Del Giudice et al., 2012) and *L. casei* (Yu et al., 2010) have been studied for their amelioration of allergic disease. Moreover, experimental studies have demonstrated the immunomodulatory role of probiotic bacteria, notably *Bifidobacterium*, in reducing the Th2 inflammation profile induced in mice (Abrahamsson et al., 2007; Iwabuchi et al., 2009; MacSharry et al., 2012). In general, the strategy has been to maintain or restore a healthy gut microbiota by regular supplementation with the abovementioned probiotics. Recently, studies have demonstrated that oral administration of probiotics protects female mice from bone density loss caused by ovary removal (Sjogren et al., 2012; Britton et al., 2014; Ohlsson et al., 2014; Parvaneh et al., 2015). Despite the role of probiotics in the Th2 inflammatory response, there are no experimental studies investigating the preventive action of probiotics on the exacerbation of asthma by low levels of sex hormones, which occurs in asthmatic women during the menstrual cycle and menopause. Considering that life expectancy has increased and that most women now undergo menopause, strategies to prevent the initiation or exacerbation of asthma in this life stage are highly relevant (Troisi et al., 1995; Gong et al., 2016). Thus, the purpose of this study was to determine whether *Bifidobacterium longum* 5^1A^ exerts a modulatory effect on the exacerbated lung inflammatory response induced in ovariectomized allergic mice. Here, we show for the first time that *B. longum* 5^1A^ administered to ovariectomized allergic mice prevented the exacerbation of lung inflammation and airway hyperresponsiveness.

## Materials and Methods

### Animals

Female Balb/c mice (18–20 g) were obtained from the animal facility of the Institute of Biomedical Sciences, University of Sao Paulo. Animals were housed in groups of five per cage in a light- and temperature-controlled room (12 h light/dark cycles, 21 ± 2°C) with free access to food (AIN93-M) (Reeves et al., 1993) and water. The local Animal Care Committee of the University of Sao Paulo Institute of Biomedical Sciences approved the experiments.

### Ovariectomy

Mice were anesthetized by an intraperitoneal (i.p.) injection of ketamine/xylazine (100 and 20 mg/kg, respectively). After an incision was made in the lower part of the abdomen, the ovaries were identified and removed from the adherent tissue. The effectiveness of the OVx procedure was assessed by analyzing the morphologic features of cells in vaginal smears and by quantifying the uterine weight. Mice subjected to similar manipulations except for the ovary removal were used as the sham-operated controls and labeled ‘sham’ animals. The same surgeon performed all surgical procedures.

### Microbial Community Profiling

Fecal samples were collected 1 day before and 10 days after surgery and fecal pellets were frozen in 2 mL tubes at -80°C until DNA extraction. Pellets were placed in MoBio PowerSoil bead tubes and incubated at 70°C for 10 min before proceeding with the protocol recommended by the manufacturer. Extracted DNA was quantified using a Qubit spectrophotometer (Thermo Scientific Technologies) and visualized in 2% agarose gels stained with ethidium bromide to evaluate DNA integrity. Microbiote analysis was performed essentially as described by Thomas et al. (2016). In summary, for PCR amplification and amplicon sequencing, the V4–V5 region of the 16S rRNA gene was amplified using the forward 5′-AYTGGGYDTAAAGNG-3′ and reverse primer 5′-CCGTCAATTCNTTTRAGTTT-3′, corresponding to the positions 562 and 906, respectively, of the *Escherichia coli* 16S rRNA gene. Three 20 μl amplification reactions were performed per sample, each containing: 2.5 μM of each primer; 10 μl of Kapa Hotstart High Fidelity Master Mix (Kapa Technologies); and 25 ng of genomic DNA (gDNA). Thermocycling conditions were: 95°C, 3 min; 98°C, 15 s; and 40°C, 30 s for 35 cycles. This was followed by a last extension step at 72°C for 5 min. Amplicons of the three reactions of each sample were pooled and purified using a MinElute PCR Purification Kit (Qiagen). The purified products were run on 1.5% agarose gels and bands within the expected amplicon range were excised using sterile and disposable scalpels and purified using the Qiaquick gel extraction kit (Qiagen) to remove artifacts, primer-dimers, and non-specific bands. Amplicons were end-repaired and Ion Torrent adaptors with barcodes were ligated using the Ion Plus Fragment Library Kit (Thermo Scientific). Equimolar amounts of barcoded-amplicons from each sample were pooled, using the Ion Torrent qPCR quantitation kit (Thermo Scientific), and used for emulsion PCR using the Ion PGM Template OT2 400 Kit (Thermo Scientific). All samples were sequenced on the Ion torrent PGM platform using a v2 chip and the Ion PGM Sequencing 400 Kit (Thermo Scientific). Sequences processed by the latest version of the Ion Torrent server (v3.6.2) were used as input into the *Qiime* (*Quantitative insights into microbial ecology*) software package (Version 1.6.0) (Caporaso et al., 2010). We removed sequences with an average quality score <20 using a 50 nt sliding window. Then, we identified barcodes used for fecal sample, allowing a maximum of two mismatches, and discarded sequences with no barcodes, and <200 nt or >500 nt after barcode removal. PCR primers identified at the start or at the end of the reads, allowing a maximum of 4 nt mismatches, were trimmed and sequences with no identifiable primers were discarded. After primer trimming, we removed all sequences below 200 nt and the remaining sequences were used as input for downstream analysis. Filtered sequences were clustered with 97% identity using UPARSE (implemented in USEARCH v7 – Edgar, 2013) and the seed sequence of each cluster was picked as a representative. Chimeric sequences (and clusters) were identified using UCHIME (Edgar et al., 2011) and the Broad Institute’s chimera slayer database (version microbiomeutil-r20110519) and excluded from further analysis. With the *Qiime* interface (default parameters), the RDP classifier (Wang et al., 2007) was used to assign a taxonomic rank to each sequence using a minimum confidence value of 80% and, subsequently, to each operational taxonomic unit (OTU). Unless otherwise stated, OTUs that occurred in fewer than 25% of all samples and with less than 3 reads were not considered. Using *Qiime*, we rarefied the OTU table to a depth of 13,851 sequences in order to calculate species richness and phylogenetic diversity, using the total number of observed OTUs and Faith’s phylogenetic diversity (Faith et al., 2009), respectively. Rarefaction analysis was performed using *Qiime* and a cutoff of 13,851 for species richness. For beta-diversity analysis, OTU-representative sequences were aligned using PyNAST (Caporaso et al., 2010) against the aligned green genes core set (DeSantis et al., 2006) using *Qiime* default parameters, and the alignments were lane-mask filtered to build a phylogenetic tree using FastTree (Price et al., 2009). Distance matrices were generated for four metrics, unweighted and weighted UniFrac (Lozupone and Knight, 2005) Bray–Curtis and Euclidean, to compare pairwise distances between the four groups using ADONIS (Oksanen et al., 2016). Due to unequal sequence sampling depth across samples, we normalized the raw number of reads for each OTU using metagenome Seq’s (Paulson et al., 2013) cumulative sum scaling function. We then used normalized counts to investigate differences between the groups at the OTU, genera, and family level.

### Probiotic Supplementation

The species of bifidobacteria (*B. longum* 5^1A^) used in this study was isolated and characterized at the Laboratory of Ecology and Physiology of Microorganisms, Institute of Biological Sciences, Federal University of Minas Gerais (Souza et al., 2013). The bifidobacteria were isolated from the feces of healthy children up to 5 years old in the city of Salvador (Bahia, Brazil) and identified by morfotintorial, respiratory, and biochemical tests, followed by multiplex PCR (Kwon et al., 2005). The bacteria were replicated in MRS broth medium (Difco) and grown under anaerobic conditions in an anaerobic jar at 37°C without stirring for 48 h. For the administration of probiotic bacteria, the mice received a daily inoculum by gavage of 0.1 mL containing 10^8^ bacterial cells, beginning 15 days before the first sensitization (when animals were 4 weeks old) and until ovariectomy for prophylactic administration and 1 day after ovariectomy for the treatment protocol.

### Induction of Allergic Lung Inflammation

Mice were sensitized intraperitoneally with 10 μg of chicken egg OVALBUMIN (OVA) grade V (Sigma Chemical Co., Saint Louis, MO, United States) dissolved in 0.2 mL of Imject alum suspension (1 mg). At days 14, 15, and 16, the mice were exposed to aerosolized OVA (1% in phosphate-buffered saline PBS) for 15 min using an ultrasonic nebulizer device (Respira Max^®^, SP, Brazil) coupled to a plastic inhalation chamber (18.5 cm × 18.5 cm × 13.5 cm). Ten days after the last challenge, the animals were subjected to ovariectomy (OVx), and 10 days later, they were re-challenged over 3 days as described above. The experimental asthma protocols were started when mice were 6 weeks old. All measurements of inflammatory parameters were performed 24 h after the last aerosol re-challenge.

### Short-Chain Fatty Acid (SCFA) Measurements

Acetic acid (99.7%) and citric acid were purchased from Sigma–Aldrich (St. Louis, MO, United States), butanol from Carlo Erba (Cornaredo, Italy), acetonitrile from Merck (Darmstadt, Germany), and tetrahydrofuran from Acros Organics (Fair Lawn, NJ, United States). Chromatographic analyses were performed using an Agilent 6850 system with EzChrom software, equipped with a 7683B automatic liquid sampler, a flame ionization detector (FID) (Agilent Technologies, United States), and a fused-silica capillary DB-23 column (Agilent Technologies, United States) with dimensions of 60 m × 0.25 mm internal diameter (i.d.) coated with a 0.15 μm thick layer of 80.2% 1-methylnaphatalene. The initial oven temperature was 100°C (hold 7 min), which was then increased to 200°C at a rate of 25°C/min (hold 5 min). The FID temperature was maintained at 260°C, and the flow rates of H2, air, and the make-up gas N2 were 30, 350, and 25 mL/min, respectively. Sample volumes of 5 μL were injected at 250°C using a split ratio of approximately 25:1. Nitrogen was used as the carrier gas at 1 mL/min (hold 4 min), reduced to 0.8 mL/min (hold 1 min) and then 0.6 mL/min (hold 1 min), and finally increased to 1 mL/min. The runtime for each analysis was 16.5 min. Acetic acid (HAc) was diluted to 1.0 mg/mL in butanol:tetrahydrofuran:acetonitrile (5:3:2), and further dilutions were made using the same solvent to generate a series of standard solutions. Blank human plasma was spiked with 1.0 mg/mL HAc. This solution was further diluted with blank plasma to prepare plasma standards of 0.015–1 mg/mL. To each tube, 40 mg of sodium chloride, 20 mg of citric acid, 40 μL of 0.1 M hydrochloric acid, and 200 μL of butanol:tetrahydrofuran:acetonitrile (5:3:2) were added. To quantify the acids, a calibration curve for the concentration range of 0.015–1 mg/mL was constructed. Probiotic culture medium (100 μL) was used to measure the acetate concentration. The concentration of plasma acetate was expressed in mM. Fecal samples were weighed into 1.5 mL tubes, crushed and homogenized in 100 mL of distilled water. Subsequently, 40 mg of sodium chloride, 20 mg of citric acid, 40 μL of 0.1 M hydrochloric acid, and 200 μL of butanol: tetrahydrofuran: acetonitrile (5:3:2) were added. The tubes were vortexed for 1 min and centrifuged at 10,000 rpm for 10 min. The supernatant was transferred to microtubes, and 5 μL was injected in triplicate into the gas chromatograph. The retention time was 7.2 min. The precision and accuracy of the assay were evaluated by analyzing samples (0.62, 0.25, and 0.75 mg/mL) in five replicates. The precision, expressed as relative standard deviation (RSD), was less than 14.34%, and the accuracy was between 87.51 and 113.56%. The standard acids, HAc, HPr, and HBu, were successfully separated in the gas-liquid chromatogram and eluted between 7 and 10 min in the temperature and gas flow program used here. The retention time was 7.2, 8.2, and 9.2 min for HAc, HPr, and HBu, respectively. The standard calibration curves showed linear relationships for all acids (*r* = 0.999). There was no interference present in the blanks at the retention time of the acids analyzed, and the analyte peaks were identifiable, discrete, and reproducible. The precision and accuracy of the assay were evaluated by analyzing samples (0.62, 0.25, and 0.75 mg/mL) in five replicates. The precision, expressed as RSD, was less than 14.34, 13.93, and 12.68%, for HAc, HPr, and HBu, respectively. The accuracy was between 87.51 and 113.56% for HAc, 91.44 and 111.60% for HPr, and 86.71 and 98.56% for HBu. The limit of quantification (LOQ) was 0.031 mg/mL for both acids analyzed, with a precision lower than 15% and accuracy within ±15%. The limit of detection (LOD) for both acids was 0.015 mg/mL, where the mean coefficient of variation (CV) value was under 20% for precision and under ±20% for accuracy. The feces extraction efficiency was 97.35 ± 5.23%. Therefore, the developed methods were shown to be reliable (Shah et al., 1991) for quantifying the SCFAs HAc, HPr, and HBu in mouse feces.

### Bronchoalveolar Lavage (BAL)

Female mice were anesthetized with ketamine/xylazine (100 and 20 mg/kg, respectively), the trachea was exposed, and a cannula was inserted. The lungs were washed three times with aliquots (each 0.8 mL) of saline injected through the cannula. From the BAL fluid (BALF), the total number of cells was counted microscopically using a Neubauer chamber. Differential cell counts were performed by cytospin analysis and prepared from aliquots of BALF (200 μL) centrifuged at 300 g for 1 min using a Citospin^®^ (Fanem). The cells were stained with Instant Prov (Newprov^@^, São Paulo, Brazil), and a total of 300 cells were counted to determine the proportion of neutrophils, eosinophils, and mononuclear cells using standard morphological criteria.

### Lung Function Analysis

All animals were anesthetized with ketamine (100 mg/kg, i.p.) and xylazine (20 mg/kg) and paralyzed with pancuronium bromide, and a stable depth of anesthesia was maintained (Ferreira et al., 2012). After tracheostomy, the trachea was cannulated with a blunt 18-gauge metal tube, and the mouse was ventilated with a computer-controlled small-animal ventilator (flexiVent; Scireq, Montreal, QC, Canada) using a tidal volume of 10 mL/kg and a respiratory frequency of 150 breaths/min. A positive end-expiratory pressure (PEEP) of 2 cm H_2_O was applied throughout. An external jugular vein was isolated for an intravenous (i.v.) infusion of methacholine (MCh). At the outset, 6 μg of MCh was provided intravenously to ensure that the animal was indeed responsive to MCh and that airway resistance returned to the baseline value after its MCh-induced rise, which indicated that the mouse was in stable physiological condition. To obtain a dose-response curve, a bolus of MCh was then injected starting at a dose of 4 μg (200 μg/mL solution in PBS; i.v. boluses of 10–40 μL). Prior to each MCh dose, the expiratory path was obstructed for 15 s to produce a deep inflation, after which exhalation was immediately allowed. Ventilation was continued for approximately 2 min between consecutive MCh doses. Airway responsiveness was equal to the Newtonian resistance (Rn) (Ferreira et al., 2012).

### Analysis of Histological Changes to Lung Tissue

Lungs were removed from mice after BAL and fixed by immersion in 4% paraformaldehyde. The lobes were sagittally sectioned, embedded in paraffin, cut into 5-μm sections, and stained with periodic acid-Schiff (PAS); mucus production was measured as previously described (Tong et al., 2006).

### Flow Cytometry

Mouse Treg cells were collected from the BALF and analyzed for CD4^+^ CD25^+^ Foxp3^+^ expression using a mouse Treg cell staining kit containing APC-labeled anti-CD4, PE-labeled anti-CD25, and FITC-labeled anti-Foxp3 (eBioscience) according to the manufacturer’s instructions. Briefly, prepared cells (1 × 10^6^) were washed by centrifugation with cold PBS, resuspended in 1 mL of fixation/permeabilization solution, and incubated in the dark at 4°C for 30–60 min. The cells were washed once with 2 mL of permeabilization buffer, collected by centrifugation, resuspended in 20 mL of blocking agent with 2% (2 mL) normal rat serum in permeabilization buffer, and incubated at 4°C for 15 min. Next, 20 mL of a fluorochrome-conjugated antibody or isotype control in permeabilization buffer was added, followed by incubation in the dark at 4°C for 30 min. Finally, the cells were washed with 2 mL of permeabilization buffer, resuspended in flow cytometry buffer (PBS with 2% FBS), and analyzed using a FACSCanto II cytometer (BD Bioscience, San Diego, CA, United States). The data were analyzed using FlowJo^®^ software.

### Quantification of Cytokine Levels in BAL and of Female Sex Hormones in Serum

Commercial preparations of paired antibodies and protein standards for measurements of mouse IL-4, IL-5, IL-10, and IFN-γ (BD Bioscience) in the BAL were used to develop ELISAs according to the manufacturer’s instructions. The determinations were performed in duplicate. Serum estradiol and progesterone concentrations were determined using an enzyme immunoassay kit according to the manufacturer’s instructions (Cayman Chemical, Ann Arbor, MI, United States).

### Statistical Analysis

The data are presented as the mean ± standard error of mean, Student’s *t*-test and ANOVA followed by a Bonferroni post-test were used for comparisons between 2 and 3 or more groups, respectively. Values of *p* ≤ 0.05 were considered significant.

## Results

### Ovariectomy Efficacy

**FIGURE 1:**
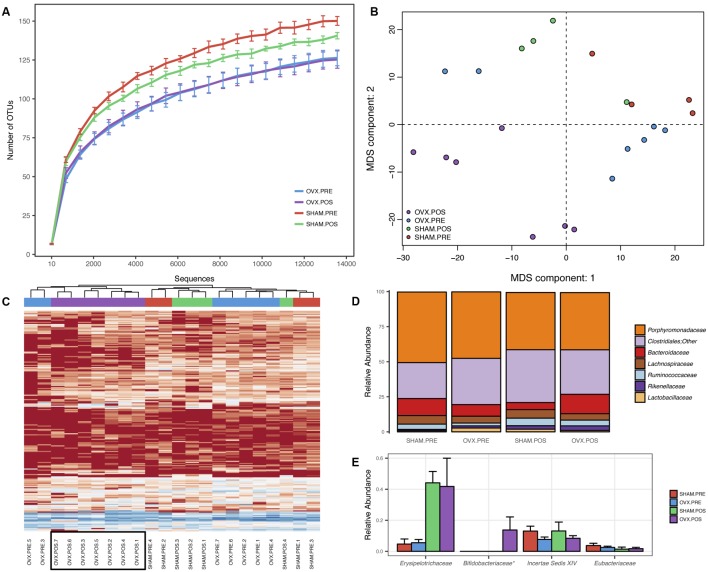
Bacterial community profiling of fecal samples from Balb/c before and after ovariectomy. **(A)** Rarefaction analysis indicating the average number of OTUs per sequence sampling depth for each of the groups. Error bars represent the standard error of the mean. **(B)** Multidimensional scaling of Euclidean distances between mouse fecal samples. **(C)** Heatmap and hierarchical clustering of mouse fecal samples using rarefied OTU abundances. **(D)** Mean relative abundances of the most abundant bacterial families identified in the samples. **(E)** Mean relative abundances of the least abundant bacterial families identified in the samples. Error bars represent the standard error of the mean and ^∗^ indicates a *p*-value < 0.05.

Ten days after ovariectomy, we measured the body and uterus weight of female mice and analyzed the vaginal cell morphology. The ovariectomized (OVx) animals had significantly higher body weights and lower uterus weights than animals subjected to false surgery (Sham). In addition, the vaginal cell morphological changes observed after ovariectomy were representative of diestrus phase and were corroborated by decreased plasma concentrations of estrogen and progesterone (**Table 1**). **Table 1** shows the parameters measured to analyze ovariectomy efficacy of animals studied in the **Figure 1**.

**Table 1:** Animal characteristics, body and tissue weights, and hormone measures.

Group	Number animals	Average body weight (g)	Uterus weight (g)	17 Beta estradiol (pg/mL)	Progesterone (ng/mL)	Diestro phase
Ovx	7	24.5 ± 1.0^∗^	0.015 ± 0.004^∗^	21.0 ± 9.9^∗^	0.78 ± 1.1^∗^	Positive
Sham	4	22.7 ± 1.0	0.028 ± 0.006	41.5 ± 3.3^∗^	1.7 ± 0.6^∗^	Negative


*Values are mean ± SD. ^∗^*p* =< 0.05 Sham vs. OVX.*

### Effect of Ovary Removal on the Gut Microbial Profile

To analyze the effect of ovariectomy on gut microbial diversity and composition, we performed 16S rRNA next-generation sequencing. We obtained a total of 1,246,075 QC-passed sequences with an average length of 329 ± 2.9 nt and an average of 56,639 sequences/sample. A total of 258 operational taxonomic units (OTUs) were obtained, which was reduced to 248 after filtering to include only those OTUs present in at least 25% of samples and with at least three reads. When analyzing species richness, we found no significant differences between groups for any of the comparisons (**Figure 1A**) but did find increased phylogenetic diversity when comparing OVx POS (post-ovariectomy) mice with Sham PRE (pre-ovariectomy) mice and OVx PRE mice with Sham PRE mice. We found significant differences in pairwise distances using the weighted UniFrac metric when we compared OVx POS mice with Sham POS mice and all groups simultaneously (**Table 2**). Multidimensional scaling (MDS) revealed that the OVx POS samples clustered farther away from the other samples (**Figure 1B**). Bacterial community composition was also investigated, revealing that the most abundant bacterial phyla were (in decreasing order of abundance) *Bacteroidetes*, *Firmicutes*, and *Proteobacteria*. Despite seeing increases in *Bacteroidetes* abundance and decreases in *Firmicutes* abundance in OVx POS mice, these were non-significant. At the family level, the most abundant bacterial families among the groups were *Porphyromonadaceae*, unclassified *Clostridiales*, *Bacteroidaceae*, *Lachnospiraceae*, and *Ruminococcaceae*. Hierarchical clustering of rarefied OTU abundances clustered all samples from the OVx POS group together in one main tree branch, whereas samples from the other groups were scattered across various branches (**Figure 1C**). When we evaluated differences between the groups at different taxonomic levels, we found a significant increase of *Bifidobacteriaceae*, but not any other bacterial taxon, in OVx POS samples (**Figure 1D**).

**Table 2:** Alpha and beta diversity *p*-values for various group comparisons.

	Metrics					
	
Comparison	Bray–Curtis	Euclidean	Unweighted unifrac	Weighted unifrac	Observed species	Phylogenetic diversity
All	0.18	0.638	**0.047**	0.517	NA	NA
OVX POS vs. SHAM POS	0.055	0.164	**0.045**	0.351	0.492	1
OVX POS vs. SHAM PRE	0.212	0.339	0.089	0.433	0.138	**0.006**
OVX PRE vs. OVX POS	0.095	0.085	0.063	0.283	1	1
OVX PRE vs. SHAM POS	0.679	0.917	0.385	0.688	0.594	1
OVX PRE vs. SHAM PRE	0.953	0.995	0.373	0.682	0.096	**0.018**
SHAM PRE vs. SHAM POS	0.164	0.589	0.056	0.358	0.168	0.312


*Bold values = *P* < 0.05.*

### Ovariectomy Exacerbates Lung Inflammation and Airway Hyperresponsiveness in Re-challenged Allergic Mice

**FIGURE 2:**
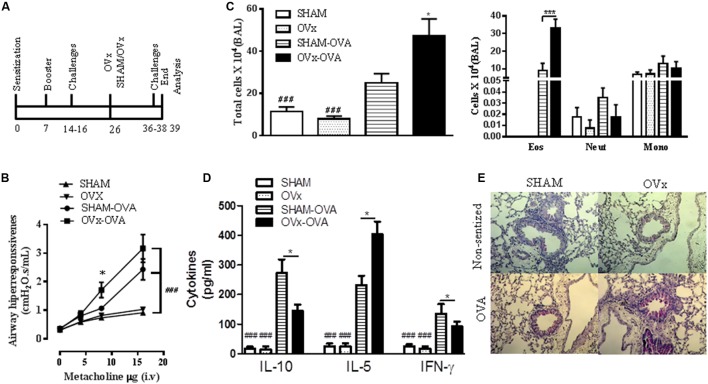
Ovariectomy exacerbates the lung inflammation and airway hyperresponsiveness of re-challenged allergic mice. **(A)** Ten days before being ovariectomized (OVx) or not (Sham), the animals were sensitized and challenged. Ten days after ovariectomy, the animals were re-challenged. All parameters were analyzed 24 h after the last re-challenge. **(B)** Measurement of AHR, as assessed by Newtonian airway resistance to increasing doses of methacholine. **(C)** Quantification of the total number of cells in the bronchoalveolar lavage (BAL). **(D)** Concentration of IL-10, IL-5, and INF-γ in the BAL. **(E)** Representative periodic acid–Schiff (PAS)-stained lung tissue from mice in the SHAM, OVx, SHAM OVA, and OVx OVA groups. Scale bars, 200 μm. All results are representative of data generated in two different experiments and are expressed as the mean ± SEM (*n* = 5 in all groups). Statistical significance was determined by a one-way analysis of variance (ANOVA), except in **(B)**, in which a two-way ANOVA test was used. Hash symbol: non-allergic (sham and OVx) groups vs. respective allergic group (sham Ova and OVx OVA), ^###^*P* < 0.001; asterisk: Sham OVA vs. OVx OVA. ^∗^*P* < 0.05, ^∗∗^*P* < 0.01, ^∗∗∗^*P* < 0.001.

We next investigated whether re-exposure of OVx allergic mice to antigen (OVA) exacerbates lung inflammation (**Figure 2A**). We found a significant increase in total cells recovered in the BALF from Sham and OVx allergic mice that was exacerbated by re-exposure of OVx allergic mice to OVA (**Figure 2C**). Moreover, the number of eosinophils in the BAL from re-challenged OVx allergic mice significantly increased compared with Sham allergic mice. The total cells in the BAL recovered from non-sensitized or OVx mice did not differ.

Next, considering that airway hyperresponsiveness is a hallmark of asthma and that its evolution is often associated with lung inflammation (Maddox and Schwartz, 2002; Tong et al., 2006), we investigated whether the ovariectomy of allergic mice would also lead to exacerbated hyperresponsiveness. We quantified the respiratory Newtonian resistance (Rn) changes in response to intravenous administration of a cholinergic agent, methacholine (MCh), after the last re-exposure to antigen challenge. Re-challenged OVx allergic mice exhibited significantly increased airway hyperresponsiveness to MCh compared with Sham OVx allergic mice (**Figure 2B**).

We also determined the effect of ovariectomy on the Th2 cytokine profile. Our data revealed a significant increase in IL-5 levels in the BAL recovered from OVx allergic mice re-exposed to antigen challenge compared with that of Sham OVx allergic mice (**Figure 2D**). Although a low level of IL-4 was found in the BALF, no significant difference between these two groups was observed (unpublished data). In contrast, IFN-γ and IL-10 were reduced in the BAL of re-challenged OVx allergic mice compared with Sham OVx allergic mice (**Figure 2D**).

Excessive mucus production is a characteristic of asthmatic airways; therefore, we evaluated the presence of PAS-stained cells in the lungs of re-challenged mice. As seen in **Figure 2E**, a significant increase in PAS-stained cells was observed in the lungs of re-challenged OVx allergic mice compared with re-challenged Sham OVx allergic mice. The lungs of non-sensitized mice did not show PAS-stained cells (**Figure 2E**).

### Preventive Oral Probiotic Supplementation Attenuates the Exacerbation of Lung Inflammation and Airway Hyperresponsiveness in Re-challenged OVx Allergic Mice

**FIGURE 3:**
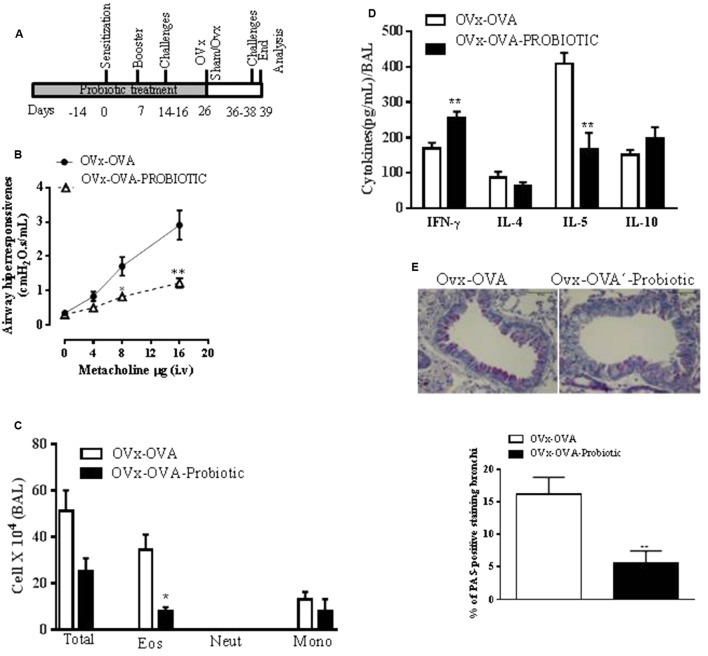
Preventive oral probiotic supplementation decreased the allergic airway inflammation and airway hyperresponsiveness of re-challenged ovariectomized allergic mice. **(A)** An oral probiotic (*B. longum* 5^1A^) was administered by gavage daily for 15 days before the first OVA sensitization of mice and continued until 4 h before the ovaries of the mice were removed. **(B)** AHR in mice supplemented with the probiotic or saline. **(C)** Differential cell counts in the BAL. Total number of cells infiltrating the airways in the BAL. **(D)** Concentration of INF-γ, IL-4, IL-5, and IL-10 expression levels in the BAL. **(E)** Representative periodic acid–Schiff (PAS)-stained lung tissue from mice on either a control or probiotic on day 16. Scale bars, 200 μm. All results are representative of data generated in two different experiments and are expressed as the mean ± SEM (*n* = 4 control mice, and *n* = 5 mice given the probiotic supplementation). Statistical significance was determined with Student’s *t*-test, except in **(B)**, in which a two-way ANOVA test was used. ^∗^*P* < 0.05, ^∗∗^*P* < 0.01, ^∗∗∗^*P* < 0.001, ^∗∗∗∗^*P* < 0.0001.

The role of probiotics in regulating the immune system and allergic diseases has been extensively debated (Abrahamsson et al., 2007). An oral probiotic (*B. longum* 5^1A^) was administered daily by gavage for 15 days before the first OVA sensitization of mice and continued until 4 h before the mouse ovaries were removed (**Figure 3A**). The preventive effect of *B. longum* 5^1A^ supplementation on the exacerbation of lung inflammation and airway hyperresponsiveness in re-challenged OVx allergic mice was investigated. Oral supplementation with *B. longum* 5^1A^ attenuated the exacerbation of the MCh-induced airway hyperreactivity of re-challenged OVx allergic mice. The airway hyperresponsiveness of supplemented OVx-OVA mice was significantly reduced compared to the OVx-OVA group. Additionally, *B. longum* 5^1A^ treatment significantly reduced the number of eosinophils recovered from the BAL of re-challenged OVx allergic mice (**Figure 3B**). *B. longum* 5^1A^ administration also resulted in a significant reduction of IL-5 levels in the BAL of re-challenged OVx allergic mice relative to non-treated mice (**Figure 3C**). By contrast, *B. longum* 5^1A^ treatment significantly increased the levels of IFN-γ in re-challenged OVx allergic mice. The BAL levels of IL-4 and IL-10 were not changed (**Figure 3D**). The probiotic treatment also significantly reduced the mucus production in the lungs of re-challenged OVx allergic mice compared with non-treated re-challenged OVx allergic mice (**Figure 3E**).

### Preventive Supplementation with *B. longum* 5^1A^ Increases the Production of SCFAs and the Number of Lung Treg Cells in Re-challenged OVx Allergic Mice

**FIGURE 4:**
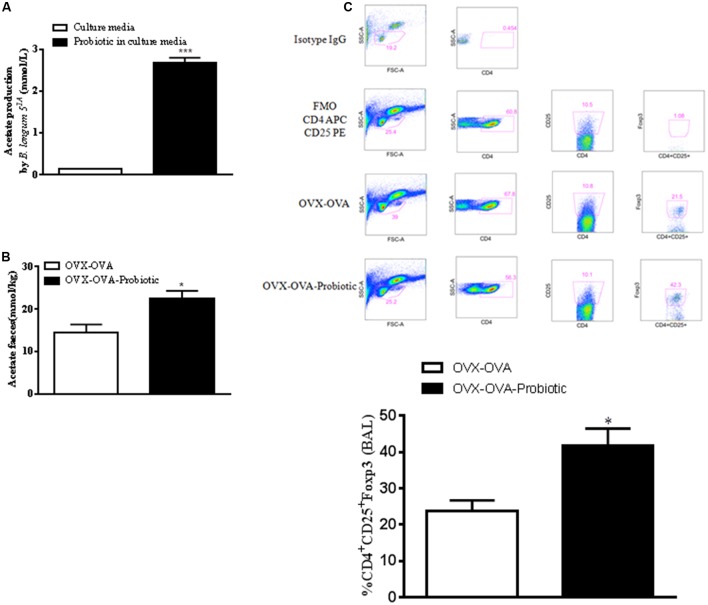
Preventive supplementation with *B. longum* 5^1A^ increases the production of short-chain fatty acids (SCFAs) and the number of lung Treg cells of re-challenged OVx allergic mice. **(A)** Sterile bacterial culture medium and *B. longum* in culture medium. Fecal SCFA concentrations from the mice depicted in **(B)**. **(C)** An oral probiotic (*B. longum* 5^1A^) was administered by gavage daily for 15 days before the first OVA sensitization of mice and continued until 24 h before the mouse ovaries were removed. After the last re-challenge, cells from the BALF were subjected to flow cytometry and stained for CD4-APC, CD25-PE, and FoxP3-FITC. The dot plot demonstrates the gate strategy of Treg (Isotype control, Fluorescence Minus One Control-FMO, OVA-OVx, and OVA-OVx-probiotic) and the percentage of positive Treg cells. The graph is representative of two independent experiments. All results are representative of data generated in two different experiments and are expressed as the mean ± SEM [*n* = 3 for all groups in **(A)**, *n* = 4 in **(B,C)**]. Statistical significance was determined by a one-way ANOVA. ^∗^*P* < 0.05, ^∗∗∗^*P* < 0.001.

Because SCFAs, especially acetate, have anti-inflammatory properties, we hypothesized that SCFA production by *B. longum* 5^1A^ could modulate allergic lung inflammation. Thus, we measured SCFAs in the probiotic culture medium and in mouse feces. Our data indicated that acetate was the SCFA quantified in the bacterial medium (**Figure 4A**), whereas we did not find measurable levels of propionate or butyrate. In addition, *B. longum* 5^1A^ treatment significantly increased the levels of acetate in the feces of re-challenged OVA allergic mice compared to non-treated mice (**Figure 4B**). Butyrate was also measured but did not differ between groups (unpublished data). Since Treg cells play a role in the control of asthma, and SCFAs interfere with the functional activity of Tregs, we examined the levels of Tregs in the BALF of the re-challenged OVx allergic mice previously treated with *B. longum.* The re-challenged OVx allergic mice supplemented with the probiotic exhibited a significant increased percentage of Tregs in BALF compared with non-treated mice (**Figure 4C**).

### Oral Probiotic Supplementation after Ovariectomy Does Not Attenuate the Exacerbated Lung Inflammation and Airway Hyperresponsiveness of Re-challenged Allergic Mice

**FIGURE 5:**
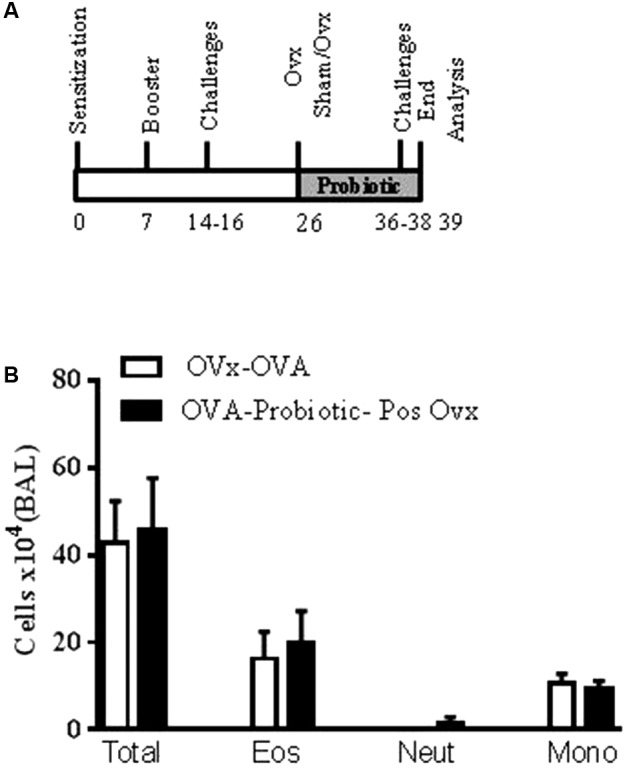
Oral probiotic supplementation after ovariectomy does not alter airway inflammation. An oral probiotic (*B. longum* 5^1A^) was administered by gavage 24 h after ovariectomy until the last re-challenge **(A)**. All parameters were analyzed 24 h after the last re-challenge. **(B)** Quantification of the total cells number in the BAL. All results are representative of data generated in one experiment and are expressed as the mean ± SEM (*n* = 4–5 mice in all groups).

In this set of experiments, we investigated the effect of probiotic supplementation on exacerbated lung inflammation and airway hyperresponsiveness after ovariectomy, when lung inflammation is established. The mice were supplemented with *B. longum* 5^1A^ after ovary removal. As shown in **Figure 5**, when the probiotic was given after ovary removal, the profile of total inflammatory cells and eosinophils recovered in the BAL was not altered in the re-challenged allergic mice relative to the non-treated re-challenged OVx allergic mice (**Figure 5**).

## Discussion

Clinical evidence shows that after puberty, asthma is more frequent in females than in males, which indicates that the oscillation of female sex hormones during the menstrual cycle is involved (Leynaert et al., 2012). In addition, asthma symptoms worsen in menopausal women (Keselman and Heller, 2015). Previous studies have investigated the beneficial effects of probiotics on allergic lung disease (Abrahamsson et al., 2007, 2009, 2011). However, the effect of probiotics on asthma symptoms or exacerbation during the perimenstrual and menopausal phases in women remains clinically debated. Many studies have shown that some Bifidobacterium levels are decreased in the presence of allergic disease (MacSharry et al., 2012). Menopause precedes senescence (Kirkwood and Shanley, 2010), and a negative correlation between senescence and Bifidobacterium use has been described in clinical studies (Arboleya et al., 2016). Additionally, *B. longum* has been associated with protection against asthma in clinical and experimental studies (Iwabuchi et al., 2009; Akay et al., 2014). The effects of *B. longum* 5^1A^ on allergic disease in particular have not been investigated yet. However, it has been demonstrated that oral *B. longum* 5^1A^ treatment resolved the inflammation due to lung infection caused by *Klebsiella pneumoniae* in mice (Vieira et al., 2016) and reduced the inflammatory response in an experimental murine model of gout (Vieira et al., 2015). Additionally, clinical studies have shown that *B. longum* 5^1A^ has an effect on pediatric functional constipation (Guerra et al., 2011).

In this study, we investigated the role of probiotic treatment of ovariectomized re-challenged allergic mice. The study was performed to evaluate the role of probiotics in asthma exacerbation during menopause. First, we confirmed that re-challenging ovariectomized allergic mice exacerbated Th2-mediated airway inflammation and airway reactivity, strongly suggesting that sex hormone deficiency after ovary removal modulates the severity of the asthma phenotype. Accordingly, after re-exposure to the antigen challenge, ovariectomized allergic mice showed exacerbated allergic inflammation, airway hyperresponsiveness, and excess secretion of mucus and the cytokine IL-5. Next, we treated the mice with *B. longum* 5^1A^ before or after the induction of allergic reaction and ovariectomy to evaluate the preventive and therapeutic effect of the probiotic in this model of allergic lung inflammation. Our data demonstrated that *B. longum* 5^1A^ prevented the exacerbation of Th2-mediated airway inflammation induced in re-challenged ovariectomized allergic mice. As the probiotic treatment began 15 days before the antigen sensitization and continued until the day of ovariectomy, we suggest that the probiotic *B. longum* 5^1A^ has a preventive effect on the exacerbation of allergic lung inflammation. In fact, our data revealed a significantly decreased eosinophil influx into the airways. In addition, reduced airway hyperresponsiveness, IL-5 levels, and mucus production were also observed. This is the first study investigating the effect of *B. longum* 5^1A^ on asthma exacerbation during menopause. Since allergic asthma is mediated by Th2 cytokines, and our data revealed an effective involvement of *B. longum* 5^1A^ in the reduction of airway hyperreactivity and eosinophil recruitment into the lungs, we hypothesized that the treatment of mice with the probiotic shifted the Th1/Th2 balance toward a Th1 profile. Indeed, in the present study, we found an association between *B. longum* 5^1A^ intake and an augmented release of the classical Th1 cytokine IFN-γ as well as decreased release of the Th2 cytokine IL-5 by the lungs of re-challenged allergic mice. Considering that *B. longum* 5^1A^ treatment increased the number of regulatory T cells recovered in the BALF of re-challenged allergic mice, we note a possible role of *B. longum* in the induction of allergen-specific tolerance or immune suppression by regulatory T cells. Our data are in line with studies (Kwon et al., 2010) showing a suppressive effect of some probiotics on Th2 cytokines with a concomitant increased stimulation of Th1 cytokines. In this context, probiotic strains such as *Lactobacilli* and *Bifidobacteria* recognizably affect regulatory T cell development, modulating the Th1/Th2 balance. Experimental and clinical evidence has shown that fermentation of fiber by microbiota or probiotics mediates the production of SCFAs, such as acetate (De Filippo et al., 2010; Tan et al., 2014; Trompette et al., 2014). To verify the interaction between probiotic intake and the generation of SCFAs in our model of re-challenged allergic mice, we measured SCFAs in the feces of these mice. We found that *B. longum* 5^1A^ intake was positively correlated with the levels of acetate in feces from re-challenged ovariectomized allergic mice. Interestingly, acetate produced by the gut microbiota has been associated with an increased number of regulatory T cells in the lungs in a murine model (Thorburn et al., 2015). It already know that mice deficient in the short chain fatty acid receptor, G-protein coupled receptor 43 (GPR43) show exacerbated asthma response. In addition, acetate can regulate regulatory T cells by interfering with gene transcription, resulting in inhibition of histone deacetylases (Tao et al., 2007; Thorburn et al., 2015). Recently, it was also described that propionate, a SCFA, promotes the development of tolerogenic DCs to the draining mesenteric lymph nodes inducing the development of Treg cells (Trompette et al., 2014). This interaction between distal organ, as lung, and gut microbiota is currently named such as the lung-gut axis (Marsland et al., 2015). Besides this interaction between pulmonary and gut immunity happen due to the production of SCFAs by the gut microbiota, the oral microbiota serves as an inoculum for the intestine (Arimatsu et al., 2014), and possible lungs (Venkataraman et al., 2015; Wu and Segal, 2017). It has suggested that lung microbiota might be transiently recolonized through microaspiration and breathing (Venkataraman et al., 2015). Thus, gut microbiota modulation can be an important strategy to treat lung diseases.

Our data show that probiotic treatment of allergic mice started 1 day after ovariectomy and maintained until the last antigen challenge did not alter the profile of inflammatory cells recruited into the lungs. Thus, protection is not given when therapies are initiated after the disease has been established. Recently, it was demonstrated that preventive rather than therapeutic treatment with a high-fiber diet attenuates clinical and inflammatory markers of acute and chronic DSS-induced colitis in mice (Silveira et al., 2017). These results suggest that any therapy aimed at modifying the gut environment (e.g., prebiotic or probiotic strategies) should be given early in the course of disease. It is also possible that the effectiveness of the probiotic *B. longum* on the control of allergic lung inflammation may involve the activity of sex hormones. There are some bacteria affected by sex hormones; for example, *Prevotella intermedia* takes up estradiol and progesterone, which enhance its growth (Kornman and Loesche, 1982). These findings deserve further investigation.

We have not investigated the specific roles of estradiol in this study, but data from the literature show that the role of estradiol in allergic disease is very complex. Data from our group show that removal of the ovaries 7 days before sensitization to OVA significantly inhibited lung eosinophilia and IL-5 levels in lung lavage fluid (de Oliveira et al., 2007). However, if ovaries are removed 1 day before sensitization to OVA, lung eosinophilia and IL-5 levels are increased (Riffo-Vasquez et al., 2007). These data suggest that sex hormone levels during sensitization are relevant to asthma. Considering that the worsening of pulmonary function observed in clinical studies during the perimenstrual and menopausal phases is a consequence of asthma, we investigated the repercussions of re-exposure to the antigen after removal of the ovaries from animals with asthma already triggered. The experimental model proposed here is novel and important for studying therapeutic alternatives to treat asthma exacerbation during the perimenstrual and menopausal phases.

We examined microbiota composition and diversity 10 days after ovariectomy and, after this period we observed no differences in the microbiota diversity between the sham and ovariectomized groups or between the pre- and post-surgery groups. However, hierarchical clustering of rarefied OTU abundances clustered all samples from the ovariectomized group together in one main tree branch, while samples from the other groups were scattered across various branches. These results suggest that the microbiota in the ovariectomized animals may have a similar composition that would cluster them separately from the other groups. Importantly, the fecal microbiome was evaluated here is a single point, and this could maybe a too short period to observe significant differences in microbiota composition.

We also observed that mice in the same experimental group showed clear variations in gut microbiota composition, though they were housed in the same facilities and treated under the same conditions. Mice with the same maternal origin but from different litters do show some differences in gut microbiota composition (Ubeda et al., 2012). The mice analyzed in our experiments were not from the same litter or mothers and maybe this could explain some of the intragroup discrepancies observed here. It is also known that male and female- derived microbiota exhibit distinct circadian rhythmicity, with females showing a more obvious oscillation than males (Liang et al., 2015). In this study, we always collected feces at the same time, in the morning, to attenuate a possible circadian influence. Another important finding was that the ovariectomized group showed increased levels of bifidobacteria. Clinical studies have shown some differences in bifidobacteria species between atopic and healthy children (Stsepetova et al., 2007). *B. adolescentis* was isolated from allergic infants, and *B. infants* and *B. bifidum* were isolated from healthy infants (He et al., 2001; Stsepetova et al., 2007; Melli et al., 2016). Studies *in vitro* and *in vivo* have shown that the ability of Bifidobacterium to stimulate the immune system is species specific (Menard et al., 2008). *B. adolescentis* is an example of a bacteria that may not regulate the immune system in some situations, and it is not an important acetate producer (Menard et al., 2008). Due to the limitations of our microbiota analyses, we did not evaluate which specific strain of bifidobacteria was increased in ovariectomized mice.

**FIGURE 6:**
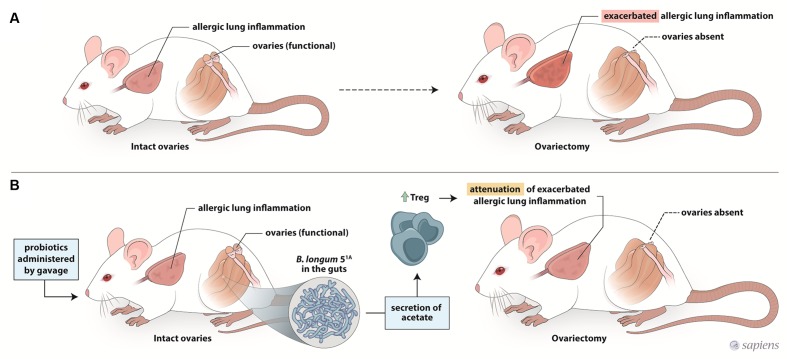
Schematic representation of mechanism underlying the effect of probiotic intake on the asthma exacerbation after ovariectomy. Ovariectomy exacerbates the lung inflammation of re-challenge allergic mice **(A)**. Preventive supplementation with *B. longum* 5^1A^ decreases exarcebation of lung inflammation and increases the production of acetate and Treg cells in re-challenge OVx allergic mice **(B)**.

In summary, our data show that the preventive supplementation of the probiotic *B. longum* 5^1A^ clearly attenuated the exacerbated airway inflammation and hyperresponsiveness in re-challenged ovariectomized allergic mice (**Figure 6**). However, the probiotic protection was lost when treatment was initiated after ovariectomy and disease was already established. These results suggest that therapeutic strategies to modulate the gut microbiota have the potential to prevent asthma exacerbation in the perimenstrual and menopausal phases. However, these strategies should be implemented before sex hormone levels decline.

## Availability of Supporting Data

Nucleotide sequences used for this study have been deposited in the SRA under accession, SRP105204.

## Author Contributions

Conceived and designed the experiments: CF, WT-L, ED-N, RC, and NC. Performed the experiments: EM, BA, AT, AF, AC, GM, and CF. Analyzed the data: EM, CF, TB, JS, and AT. Contributed reagents/materials/analysis tools: CV, WR, RC, and FM. Wrote the paper: CF, WT-L, ED-N, and RC.

## Conflict of Interest Statement

The authors declare that the research was conducted in the absence of any commercial or financial relationships that could be construed as a potential conflict of interest.
